# Supplementary material for: Molecular basis for the assembly of the Vps5-Vps17 SNX-BAR proteins with Retromer
Source: Nat Commun. 2025 Apr 15;16:3568. doi: 10.1038/s41467-025-58846-8 (PMC12000511; doi:10.1038/s41467-025-58846-8)

SUPPLEMENTARY INFORMATION

Molecular basis for the assembly of the Vps5-Vps17 SNX-BAR proteins with Retromer

Kai-En Chen[1], Vikas A. Tillu[1], Navin Gopaldass[2], Sudeshna Roy Chowdhury[2], Natalya Leneva[3], Oleksiy Kovtun[3], Juanfang Ruan[4], Qian Guo[1], Nicholas Ariotti[1], Andreas Mayer[2], Brett M. Collins[1*]

[1]Institute for Molecular Bioscience, the University of Queensland, St Lucia, Queensland, 4072, Australia.

[2]Department of Immunobiology, University of Lausanne, Epalinges, 1066, Switzerland.

[3]Research Group Molecular Mechanism of Membrane Trafficking, Max Planck Institute for Multidisciplinary Sciences, Göttingen, 37077, Germany.

[4]Electron Microscope Unit, Mark Wainwright Analytical Centre, University of New South Wales, Sydney, New South Wales 2052, Australia.

*__Corresponding author__: Brett M. Collins, b.collins@imb.uq.edu.au

This file contains 13 supplementary figures and figure legends and 4 supplementary tables

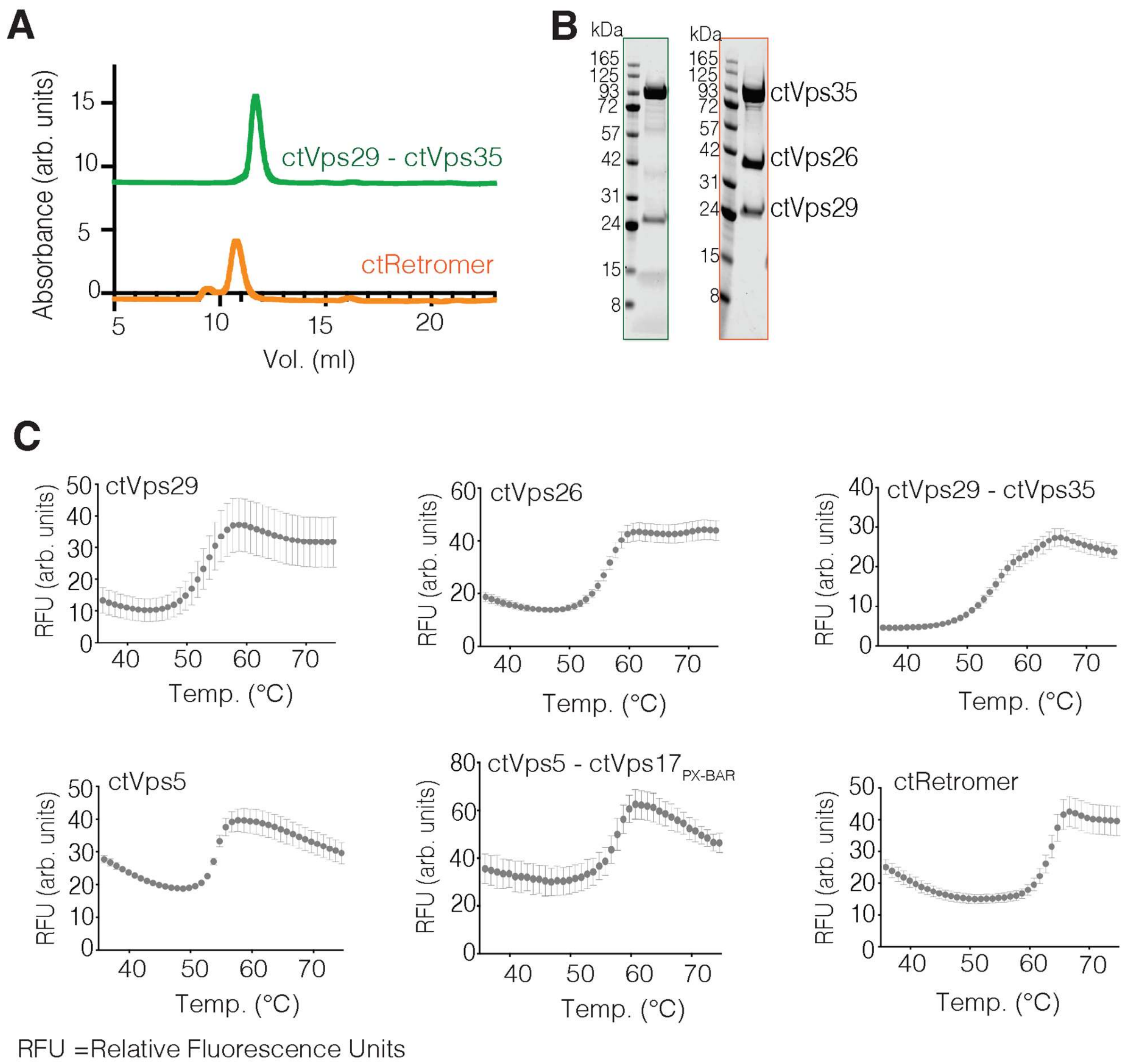

Supplementary Figure 1. Integrity and purify of ctRetromer and ctVps29–ctVps35 used in this study.

(A) Typical size-exclusion chromatogram of ctRetromer and ctVps29 – ctVps35 subcomplex purified using S200 10/300 column. Vol. indicates volume. **(B)** Associated SDS-PAGE gel showing the purity and integrity of ctRetromer and the subcomplex. **(C)** Differential scanning fluorimetry showing the folding status of the purified ctVps5, ctVps5–ctVps17$_{PX\text{-}BAR}$, ctRetromer and the associated subunits. Temp. indicates temperature.

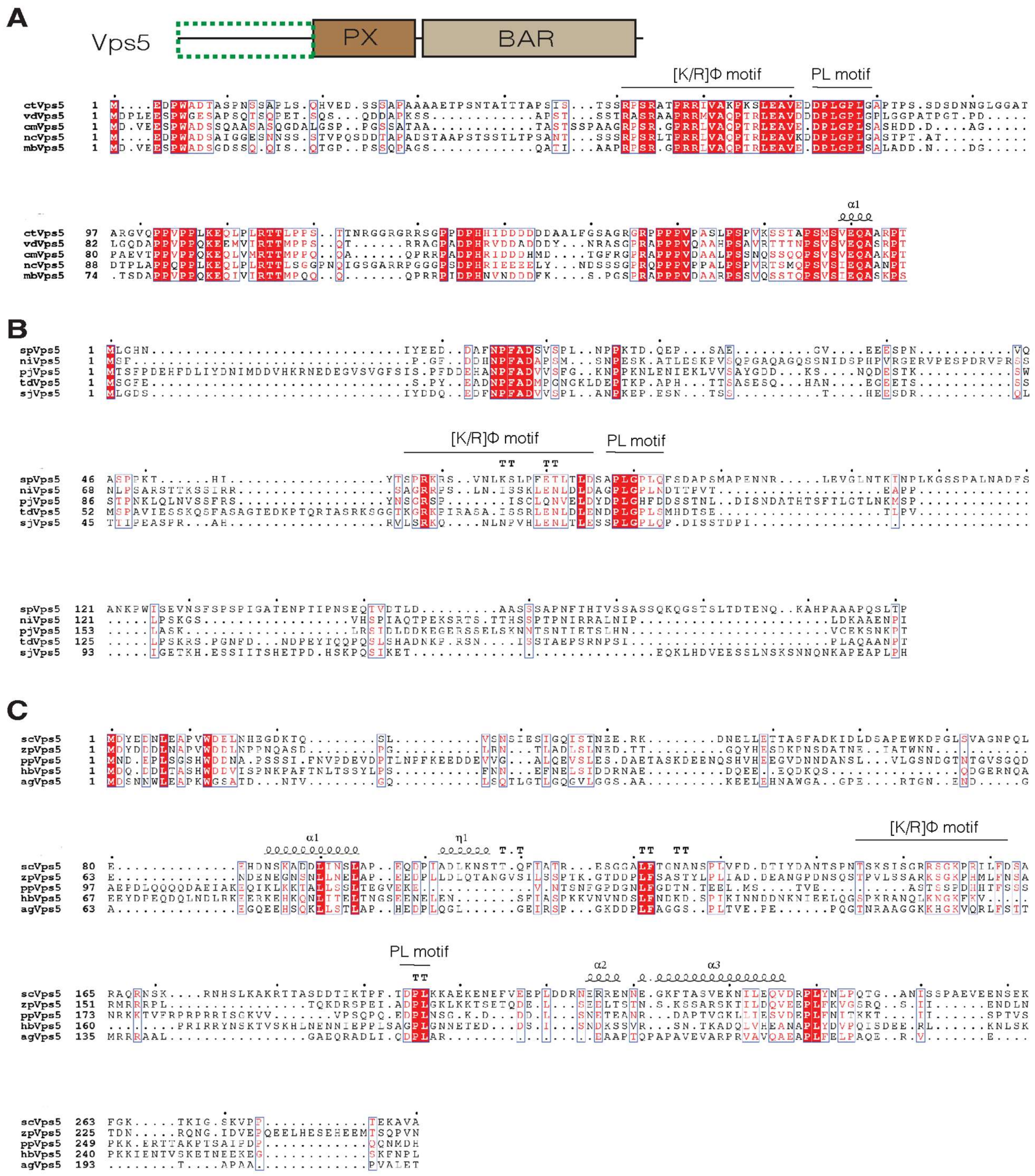

Supplementary Figure 2. Sequence alignment of yeast and fungus Vps5.

(A) Sequence alignment of Vps5 unstructured N-terminal domain across Pezizomycotina, **(B)** Schizosaccharomycetes, and **(C)** Saccharomycotina subdivisions. *ct, Chaetomium thermophilum; vd, Verticillium dahlia*; *cm, Cordyceps militaris; nc, Neurospora crassa; mb, Metarhizium brunneum; sp, Saccharomyces pombe; ni, Neolecta irregularis; pj, Pneumocystis jirovecii; td, Taphrina deformans; sj, Schizosaccharomyces japonicus*, *Sc, Saccharomyces cerevisiae; zp,* Zygosaccharomyces parabailii*; pp, Pichia_Pastoris; hb, Hyphopichia burtonii; Ag, Ashbya gossypii.*

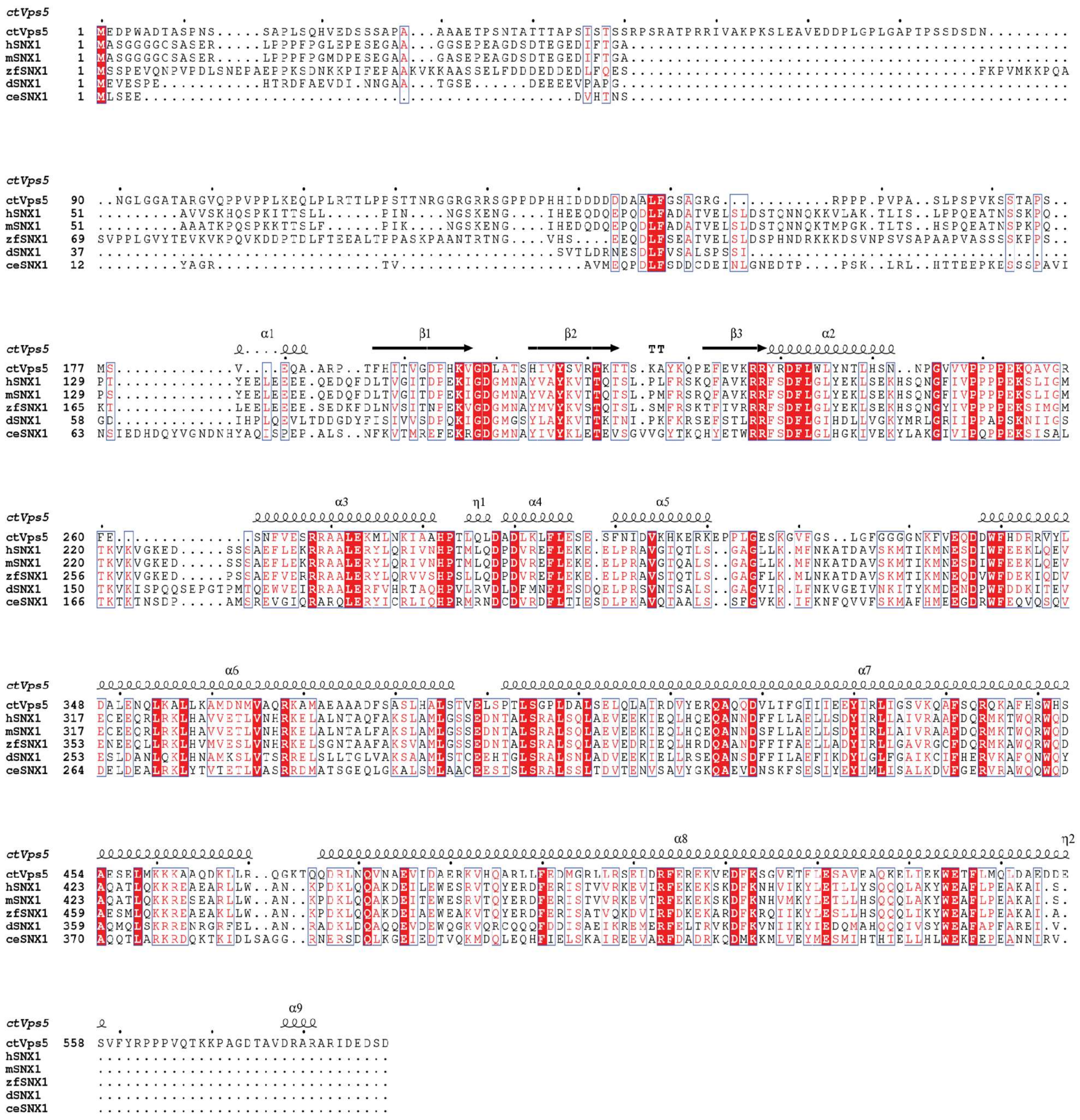

Supplementary Figure 3. Sequence alignment of ctVps5 and SNX1 from higher eukaryotic species.

Ct, Chaetomium thermophilum; h, homo sapiens; m, Mus musculus; zf, Zebrafish; d, Danio rerio; Ce, Caenorhabditis elegans.

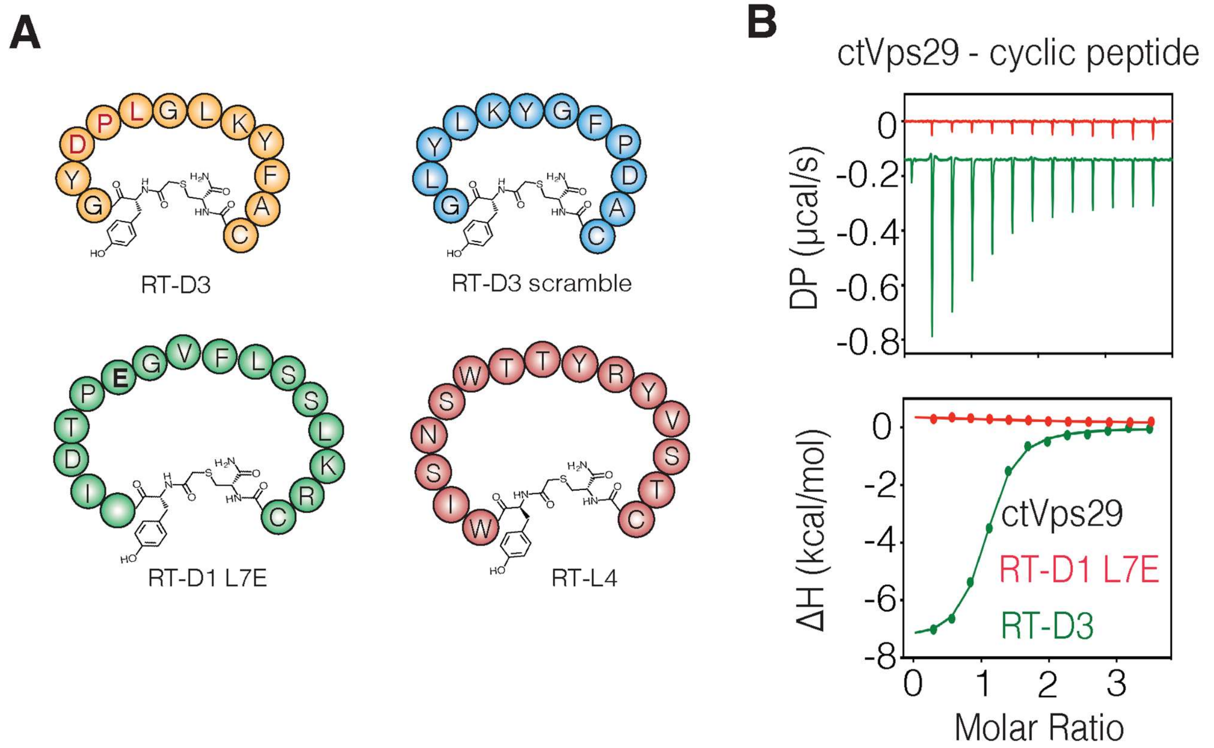

Supplementary Figure 4. Effect of the cyclic peptide on ctVps29.

(A) Schematic diagram of the four different cyclic peptides previously identified that applied in this study. RT-D3 contains PL motif (highlight in red) capable of binding to Vps29 and inhibits PL motif-containing proteins such as Vps5 from binding. RT-D1 (L7E) and RT-D3 scrambles are unable to interact with Retromer/Vps29 due to loss of the core PL sequence. Molecular stapler RT-L4 binds to the Vps26 – Vps35 interface. **(B)** Binding of previously identified macrocyclic peptides to ctVps29. ITC graph shows the integrated and normalized data fit with a 1 to 1 binding ratio.

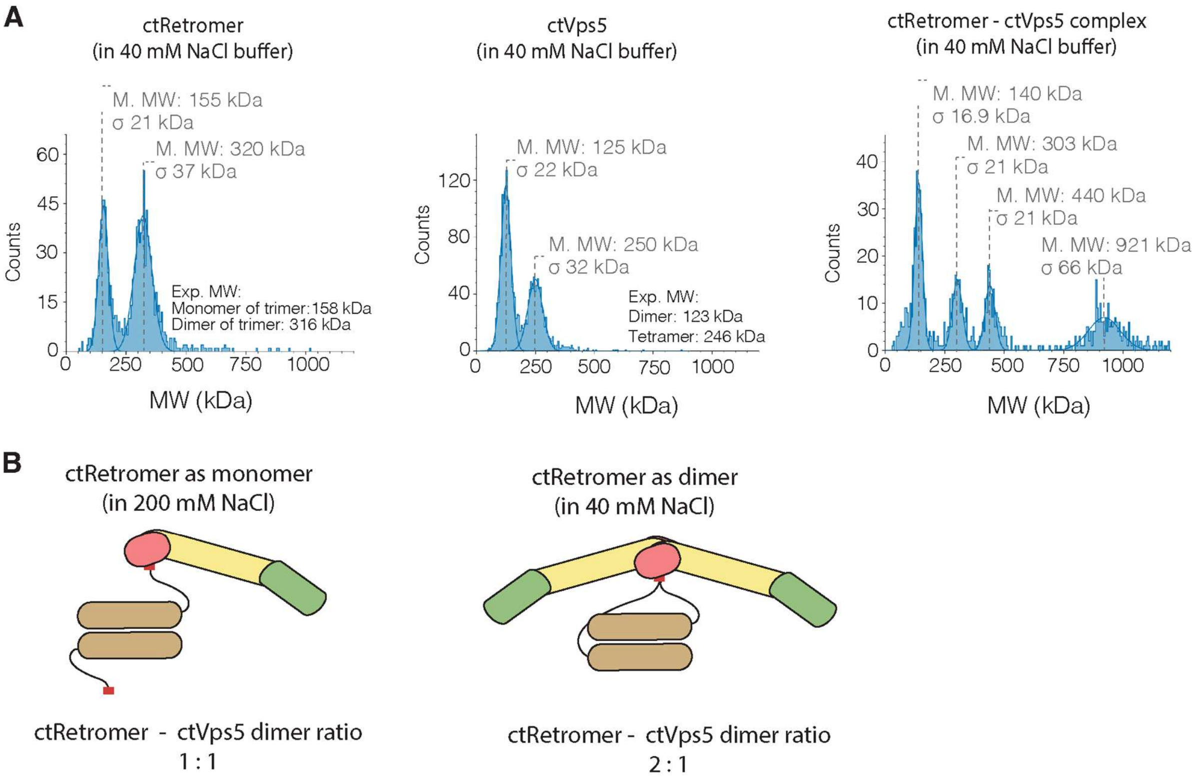

Supplementary Figure 5. ctVps5-ctRetromer complex formation in low ionic strength condition.

(A) Mass photometry of ctRetromer, ctVps5 and the ctVps5–ctRetromer complex in buffer containing 40 mM NaCl. **(B)** Schematic diagram showing the formation of 1 to 1 or 2 to 1 ctRetromer–ctVps5 dimer ratio complex in solution depending on the ionic strength of the buffer.

A

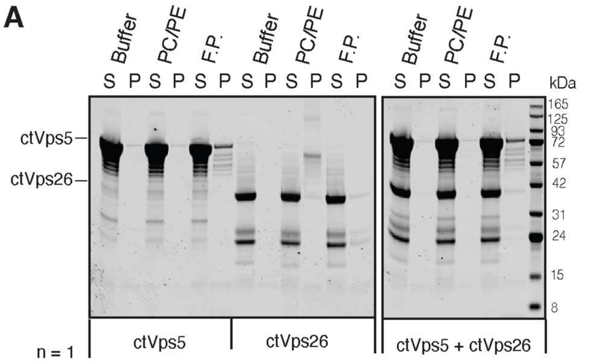

F.P. = Folch I + PtdIns(3)*P* liposome extruded using 0.4 μm pore-size membrane.

B

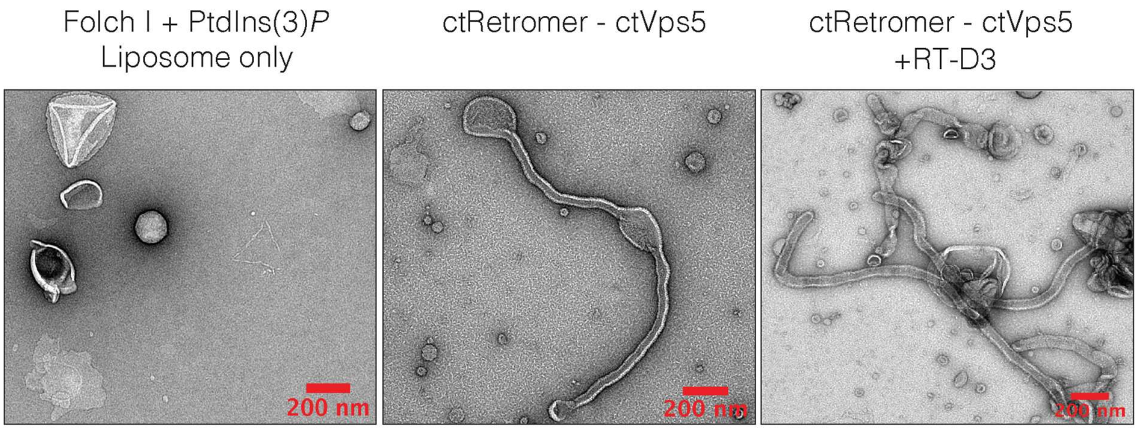

Supplementary Figure 6. The importance of ctVps5 PL motif in recruiting ctRetromer to the membrane.

(A) Liposome-pelleting assay of ctVps26 and ctVps5. In the absence of ctVps29 and ctVps35 subunits, ctVps26 alone fails to interact with ctVps5 in the presence of Folch I lipids supplemented with PtdIns(3)*P*. “S” and “P” indicates unbound supernatant and bound pellet respectively. **(B)** Negative staining images of ctRetromer–ctVps5 complex in the presence (right image) and absence (middle image) of cyclic peptide RT-D3. The image on the left shows a typical Folch I liposome supplemented with PtdIns(3)*P*, extruded using a 400 nm pore-size membrane.

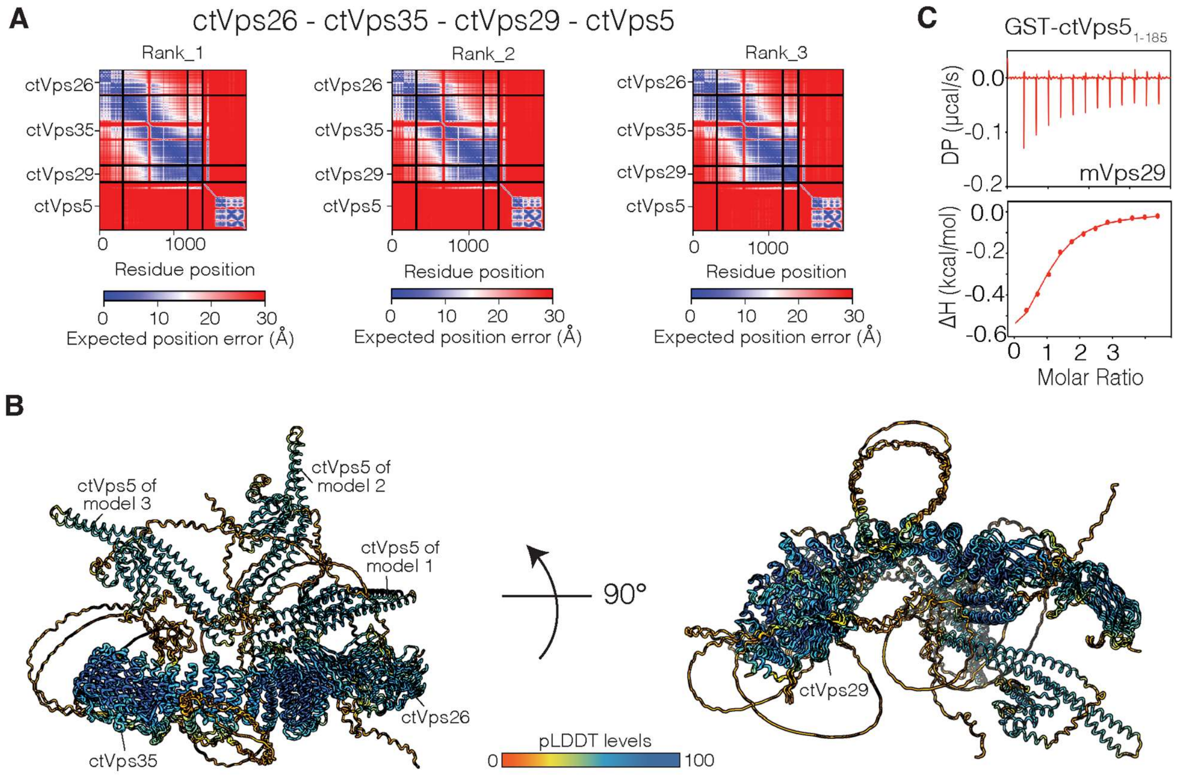

Supplementary Figure 7. AlphaFold2 predictions of ctRetromer in complex with ctVps5 highlighting the conservation of the PL motif mediated binding mechanism.

(A) Predicted alignment Error (PAE) plots showing the three predicted models of ctVps26–ctVps35–ctVps29–ctVps5 complex. **(B)** Superposition of the three representative models generated by Alphafold2. Models were colored according to the pLDDT score. **(C)** ITC measurement showing mVPS29 is capable of binding to GST-tagged ctVps5$_{1\text{-}185}$. ITC graph shows the integrated and normalized data fit with a 1 to 1 binding ratio.

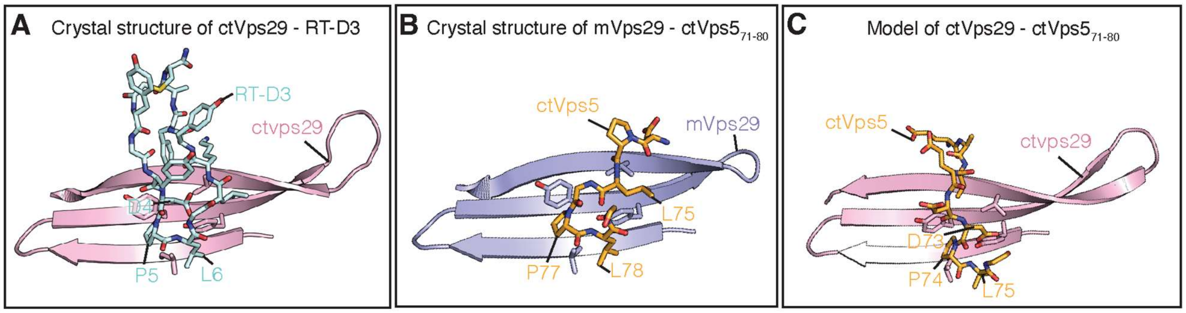

Supplementary Figure 8. Comparison of the ctVps5$_{71\text{-}80}$ peptide and RT-D3 bound Vps29 models.

(A) Cartoon representation of the ctVps29–RT-D3 crystal structure (PDB ID: 6XS8), (**B**) mVps29–ctVps5$_{71\text{-}80}$ peptide crystal structure (this study), and (**C**) ctVps29–ctVps5 Alphafold2 model. For clarity, only conserved hydrophobic surface of Vps29 and the key interacting residues are shown.

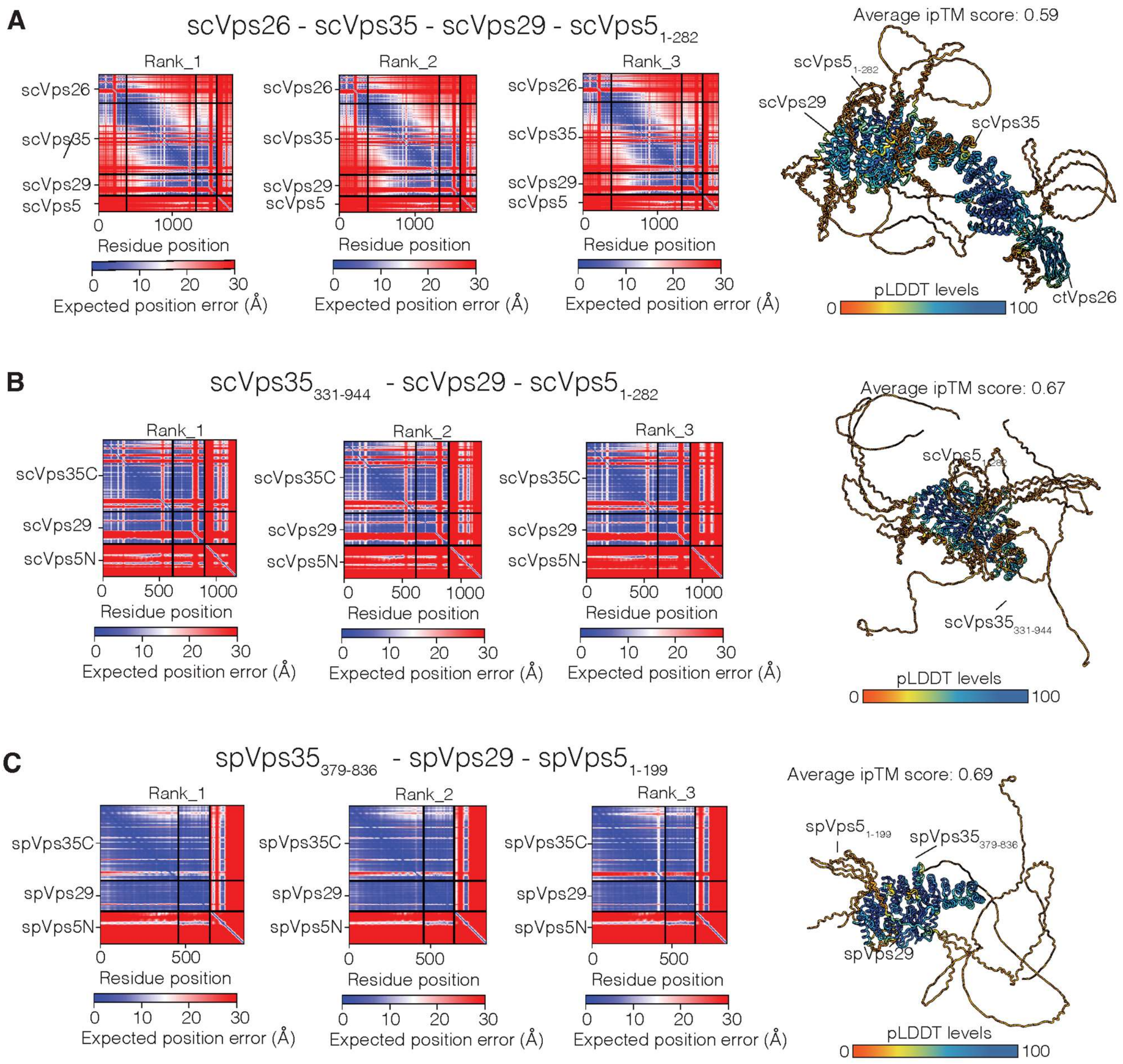

Supplementary Figure 9. AlphaFold2 predictions of Retromer in complex with Vps5 from two different yeast species.

(A) Right panel presents the PAE plots of the three predicted scRetromer–scVps5 models, **(B)** scVps35$_{331-944}$–scVps29–scVps5$_{1-282}$ models, and **(C)** spVps35$_{379-836}$–spVps29–spVps5$_{1-199}$ models. Next to the PAE plot is the superposition of the three top ranked predicted complexes colored according to the pLDDT score.

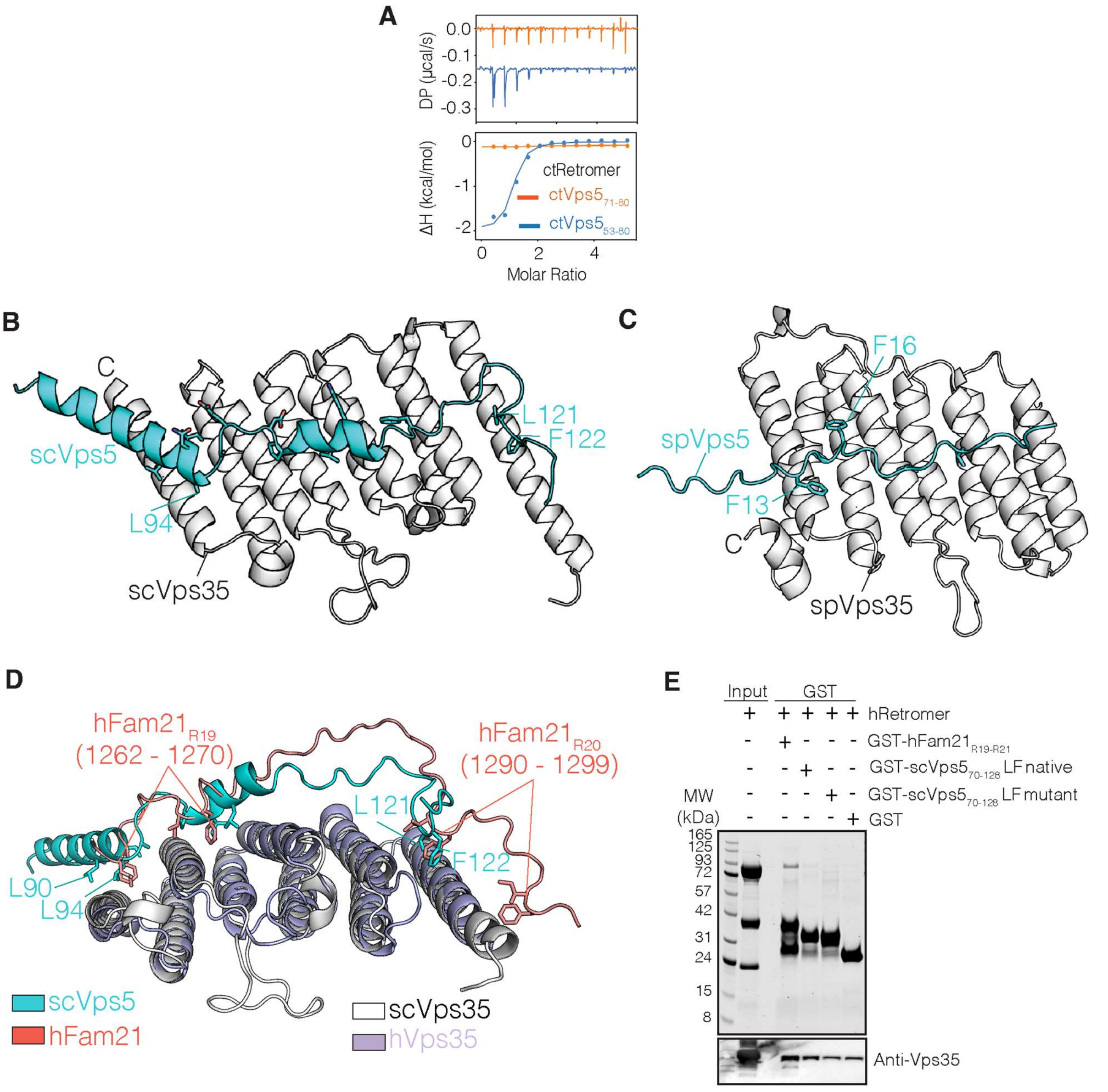

Supplementary Figure 10. Extended Vps35 binding sites observed in AlphaFold2 models of Retromer in complex with Vps5 from *S. cerevisiae* and *S. pombe*.

(A) ITC measurements show that ctRetromer binds strongly to the ctVps5$_{53-80}$ peptide (long), which contains both [K/R]Φ and PL motif, compared to the PL motif only ctVps5$_{71-80}$ peptide (short). ITC graph shows the integrated and normalized data fit with a 1 to 1 binding ratio. **(B)** Representative (top-ranked) predicted model of scVps35$_{331-944}$–scVps29–scVps5$_{1-282}$ complex, and **(C)** spVps35$_{379-836}$–spVps29–spVps5$_{1-199}$ complex. In both cases only the C-terminal region of Vps35 and the key residues of Vps5 involved in binding are shown. **(D)** Superimposition of the scVps35- scVps29–scVps5 model with the hVps35-hVps29-hFam21R19-R20 model highlights the conservation of the Vps35 binding site. For clarity, only the C-terminal region of Vps35 and the key binding residues are

shown. (**E)** GST pull-down showing the weak interaction between GST-tagged scVps5$_{70\text{-}126}$ LF motif containing loop and hRetromer. GST-hFAM21$_{R19\text{-}R21}$ was used as the positive control.

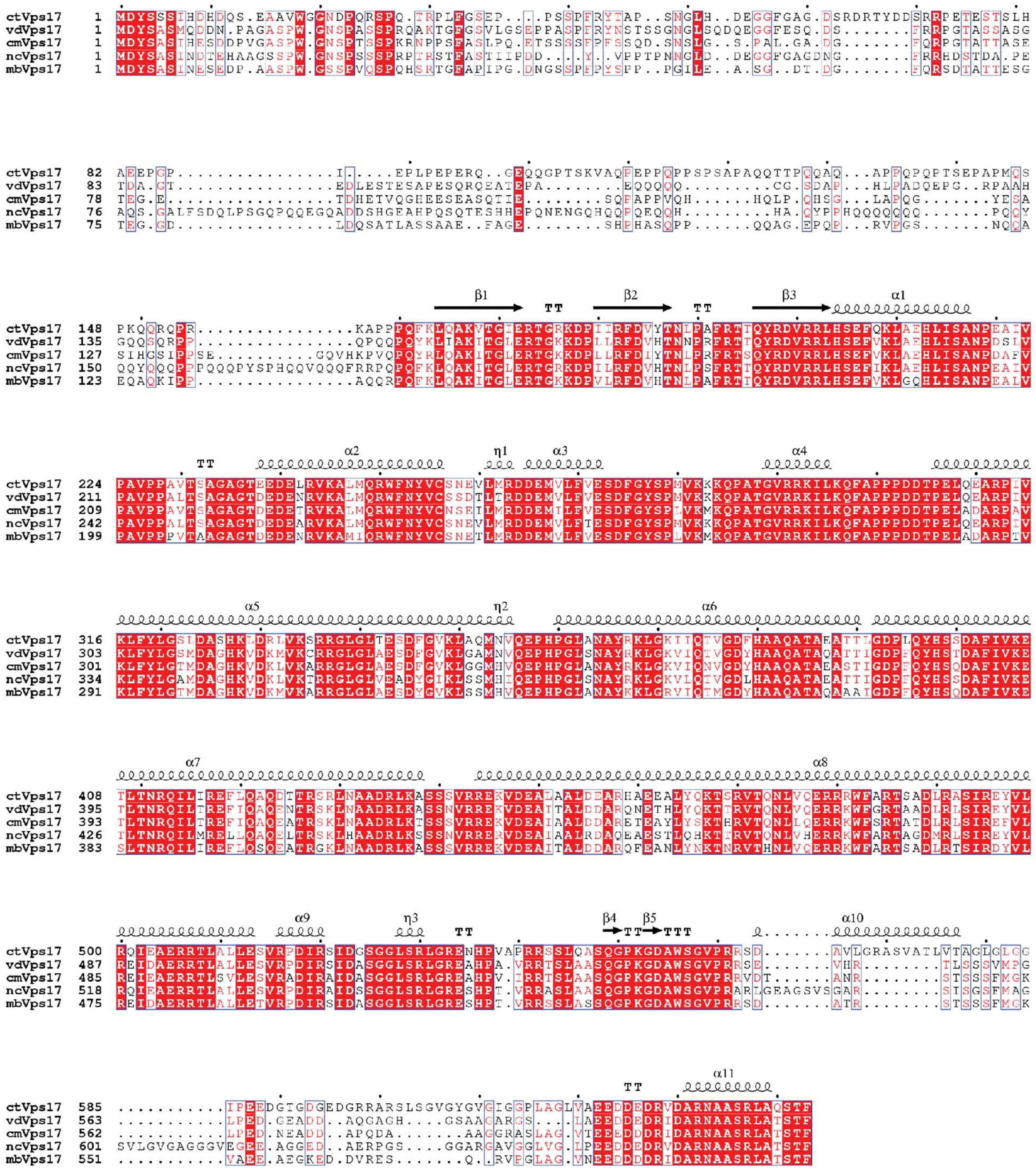

Supplementary Figure 11. Sequence alignment of Vps17 across the Pezizomycotina subdivision.

The N-terminal disordered region of Vps17 is highly divergent in sequence compared to the PX and BAR domains. *ct, Chaetomium thermophilum; vd, Verticillium dahlia*; *cm, Cordyceps militaris; nc, Neurospora crassa; mb, Metarhizium brunneum.*

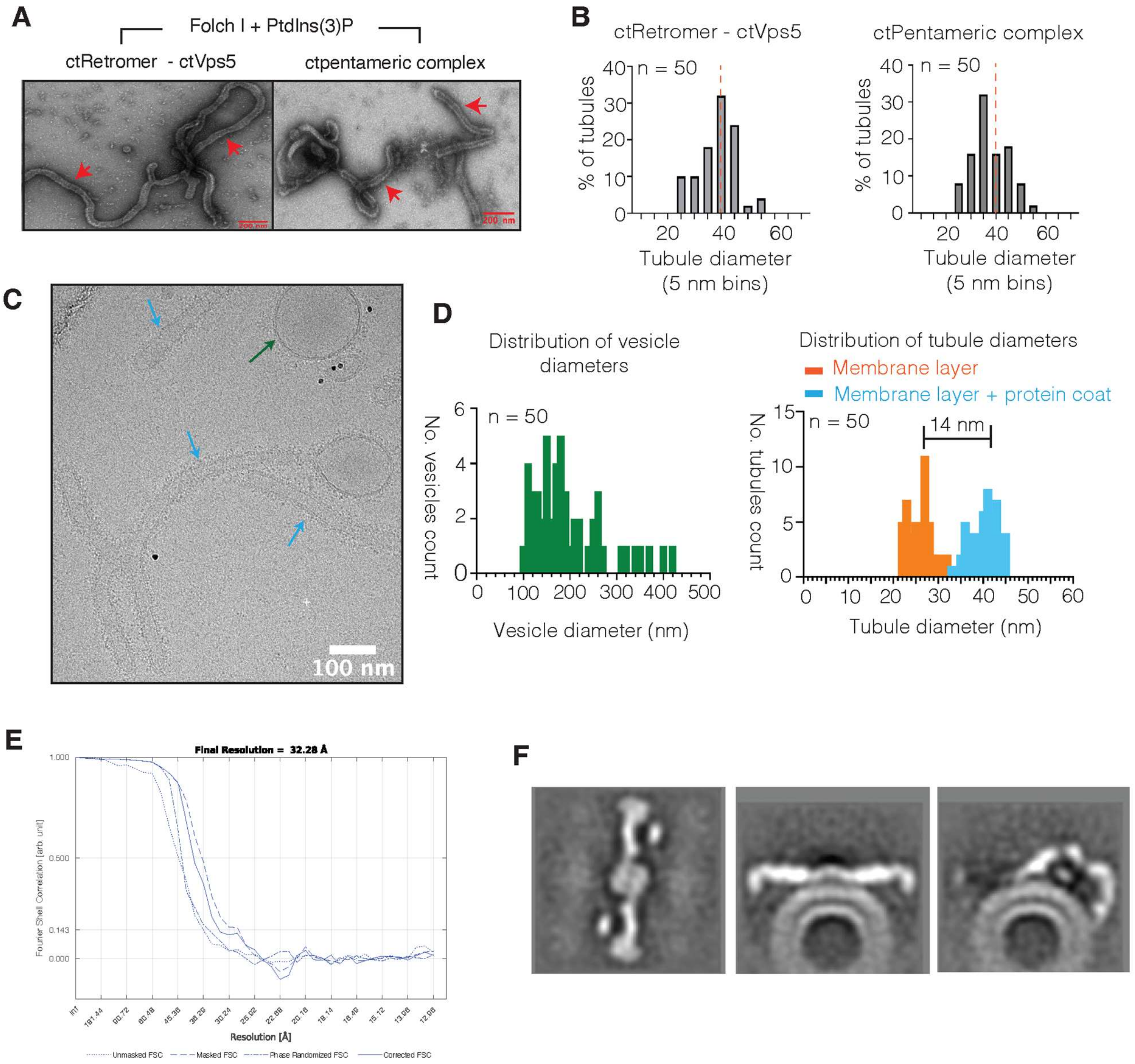

Supplementary Figure 12. Analysis of Retromer tubules by negative stain EM and cryoEM.

(**A**) Example micrographs of negatively stained Folch I - PtdIns(3)*P* liposomes incubated with ctRetromer–ctVps5 complex (left) or ctRetromer–ctVps5–ctVps17PX-BAR complex (right). (**B**) Histogram showing the diameter of tubules generated by ctRetromer–ctVps5 or ctRetromer–ctVps5–ctVps17$_{\text{PX-BAR}}$ pentameric complexes binned as percentage of total number of tubules. **(C)** Example

CryoET micrograph of ctRetromer–ctVps5–ctvps17$_{\text{PX-BAR}}$ coated tubules at 0° tilt. **(D)** Histogram showing the diameter of vesicles (left) and tubules (right) generated by the pentameric complex. For the tubules, two different measurements were carried out. The histogram highlighted in orange indicates the measured distance from one side of the membrane to another. The measurement in light blue indicates the distance from the apex region of the observed protein coat from one side to the other side of the membrane layer. For all measurements, a total of 50 tubules and vesicles were

randomly selected from the collected tilt series. (**E**) FSC of two independently processed half maps. (**F**) Orthoslices through the final sharpened and filtered to EM map, sharpened with B -2500.

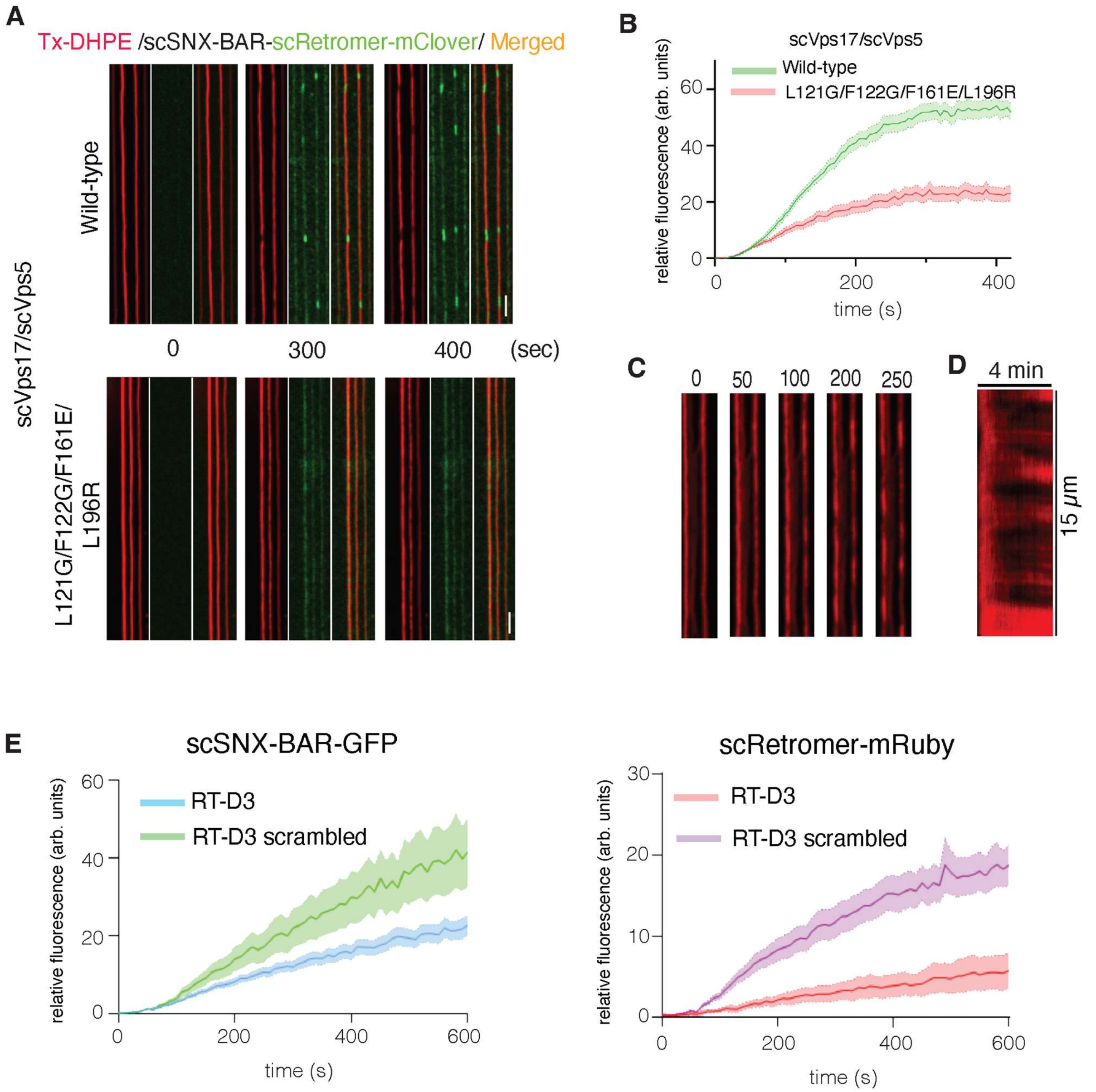

Supplementary Figure 13. Effect of scVps5 mutant on scRetromer recruitment on supported membrane tubes.

(A) Effect of the scVps5 mutant (L121G/F122G/F161E/L196R) on the recruitment of scRetromer-mClover to supported membrane tubes (SMTs). SMTs containing 5% PtdIns(3)*P* were formed and incubated with 25 nM scSNX-BAR and 25 nM scRetromer-mClover while being imaged by confocal microscopy. Frame rate: 0.2 Hz. Scale bar 2 µm. Top scVps17/scVps5 wild-type, bottom mutant. See also Supplementary Movie 7, 8. **(B)** Quantification of the recruitment of scRetromer-GFP on the tube. Quantification was done using FIJI. Measuring the mean fluorescence signal on the tube over a 10 µM length (N=13 (WT) and N=14 (mutant) tubes from 2 experiments. **(C)** SMTs were incubated with 100 nM scSNX-BARs and imaged by confocal microscopy at a fame rate of 0.2 Hz. Red: Texas-Red

DHPE. **(D)** Kymograph of SMT shown in (C). **(E)** Quantification of the scSNX-BAR-GFP and scRetromer-mRuby recruitment to SMTs. The graph shows the replot of Figure 6C with scSNX-BAR-GFP (left) and scRetromer-mRuby (right) grouped according to the presence of cyclic peptide RT-D3 or RT-D3 scrambled. Note that the lower amount of scSNX-BAR-GFP recruitment in the presence of RT-D3 was due to the lack of organised coat formation with Retromer.

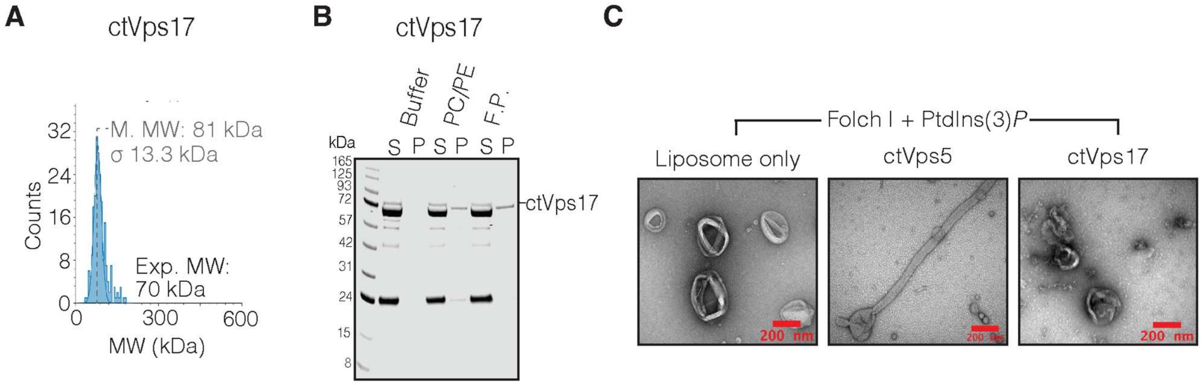

Supplementary Figure 14. ctVps17 failed to form homodimer and inability to remodel membrane in the absence of ctVps5.

(**A**) Mass photometry of full-length ctVps17. (**B**) Liposome-pelleting assays of ctVps17. Unilamellar vesicles were composed of either PC/PE control lipid, or Folch I lipids supplemented with PtdIns(3)*P*. "S" and "P" indicates unbound supernatant and bound pellet respectively. **(C)** Example micrographs of negatively stained Folch I - PtdIns(3)*P* liposome, extruded to 400 nm diameter and incubated with either ctVps5 or ctVps17.

Supplementary Table 1. Summary of crystallographic structure determination statistics

**Data collection statistics**	**mVps29 – ctVps5$_{71-80}$**
PDB ID	8FUD
Space group	C121
Resolution (Å)	42.46 – 1.68
	(1.71 – 1.68)
a, b, c (Å)	54.05, 68.60, 211.14
α, β, γ (°)	90.00, 90.00, 90.00
Total observations	332,485 (15,268)
Unique reflections	44,804 (2,057)
Completeness (%)	98.9 (91.4)
$R_{merge}{}^{+}$	0.043 (0.301)
$R_{pim^{*}}$	0.017 (0.116)
CC1/2	0.999 (0.972)
$\langle I/\sigma(I)\rangle$	18.6 (4.0)
Multiplicity	7.4 (7.4)
Molecule/asym	2
**Refinement statistics**	
R_{work}/R_{free} (%) ¶#	17.7/20.3
No. protein atoms	3189
Waters	230
Wilson B (Å^2)	21.74
Average B (Å^2)^	27.4
Protein	26.3
peptide	39.9
Water	35.5
rmsd bonds (Å)	0.007
rmsd angles (°)	1.068
Ramachandran plot:	
Favored/outliers (%)	98.4/1.1

Values in parentheses refer to the highest resolution shell. $^{+}R_{merge} = \Sigma|I - \langle I\rangle| / \Sigma\langle I\rangle$, where I is the intensity of each individual reflection. $^{*}R_{pim}$ indicates all I^{+} & I^{-}. $^{¶}R_{work} = \Sigma h|F_o - F_c| / \Sigma_l|F_o|$, where F_o *and* F_c are the observed and calculated structure-factor amplitudes for each reflection *h.* $^{\#}R_{free}$ was calculated with 10% of the diffraction data selected randomly and excluded from refinement. ^Calculated using Baverage.

Supplementary Table 2: CryoET data collection and processing statistics

**Data collection (units)**	**ctRetromer – ctVp5 – ctVps17$_{PX\text{-}BAR}$**
Magnification	45,000
Detector	Falcon 3EC
Voltage (keV)	200
Electron exposure (e/ Å^2)	120 - 130
Defocus range (µm)	-3.5 to -5.0
Pixel size (Å)	3.24
Energy filter	Yes
Slit width (eV)	20
Tilt range (min/max, step)	-60 ° /+60 °, 3 °
Tilt scheme	Dose-symmetrical
Symmetry imposed	No

Supplementary Table 3: Yeast strains used in this study

Strain	Genotype
SEY6210	∆vps5 Vam10-V5::LEU2 Vps10^{mNeonGreen}
SEY6210	∆vps5 Vam10-V5::LEU2 Vps10^{mNeonGreen} , plasmid pRS314-VPS5-HA::TRP1
SEY6210	∆vps5 Vam10-V5::LEU2 Vps10^{mNeonGreen} , plasmid pRS314-VPS5$^{\Delta 1\text{-}280}$-HA::TRP1
SEY6210	∆vps5 Vam10-V5::LEU2 Vps10^{mNeonGreen} , plasmid pRS314-VPS5^{L196R}-HA::TRP1
SEY6210	∆vps5 Vam10-V5::LEU2 Vps10^{mNeonGreen}, plasmid pRS314-VPS5$^{L196R\ L121G\ F122G\ F161E}$-HA::TRP1
NG501	CUY9228 Δpep4
NG502	NG501 Vps5 L121G F122G F161E L196R

Supplementary Table 4: Primers used in the yeast study

Primers	Sequence 5' -> 3'
RC71_Fw guide Vps5	*ATCACTGCCCAAGTACAACGGTTTTAGAG*
RC72_Rv guide Vps5	*CTAGCTCTAAAACCGTTGTACTTGGGCAGTGATACGT*
RC75_ FwVps5del	*GGGATTTTATAAACTTTCATACATCCTGCAATAACAAGCCATG*
	ATCTGTATACAATAACTTTATGTGTCACGTTCCTCAG
RC76_ R Vps5 del	*CTGAGGAACGTGACACATAAAGTTATTGTATACAGATCATGG*
	CTTGTTATTGCAGGATGTATGAAAGTTTATAAAATCCC
RC77_F Vps5 chk up	*GTCCGCTAAGAACAACTAAG*
RC78_R Vps5 chk dw	*GGATATTGAAATGCTTCTGAGC*
RC57_Pr Vam10- F-NotI	*tctaaGCGGCCGCCGCCTGCTTACCTGTGC*
RC63_Vam10-R-Sma1	*aatctCCCGGGAATATCTACAAATGAAGAAAGAAAAGAC*
RC64_pCM189-V5-CYC1-Sma1-F	*aatctCCCGGGGGCAAGCCCATCCCCAAC*
RC65_pCM189-V5-CYC1 (HindIII)-R	*CATGATTACGCCAAGCTTGGCC*
RC66_Kpn1-F-Vps5 Pr	*catctGGTACCCTGTACTGAAGTAGTCAGGAATC*
RC67_Sma1-R-Vps5	*aatctCCCGGGAAGATTGGTTTGGTAGAATGTCTC*
RC68_Sma1-F-HA-ADH1 Tr	*aatctCCCGGGggtagtgctggaagtgca*
RC69_mNG-HA-ADH1 Tr-R-Spe1	*caggcACTAGTccctgttatccctagcgg*
RC70_N-ter Del_Vps5 -Nco1F	*aatctCCATGGctTTCAAAGTTGAAGTTAAAGAC*
RC79_Vam10 start Codon Mut-F	*TCACCAAATACTTCAAAAAGtATCTCTGGAAGAAGATCCGG*
RC80_Vam10 start Codon Mut-R	*CCGGATCTTCTTCCAGAGATaCTTTTTGAAGTATTTGGTGA*
RC81_Vps5 Mut-L196R-F	*CACCCTTTACTGATCCCagaAAGAAAGCTGAAAAAGAG*
RC82_Vps5 Mut-L196R-R	*CTCTTTTTCAGCTTTCTTtctGGGATCAGTAAAGGGTG*
pNG624 Guide Vps5 PL F	*TCTTTTTCAGCT TTCTTTAAGTTTTAGAG*
pNG624b Vps5 Nterm-rev	*CATTCCTATCGTCCAATGGCTCTTC*
pNG628 Guide Vps5 PL F_2	*TCC AGT GAA CAA TGC GCC ACG TTT TAG AG*
pNG629 Guide Vps5 PL R_2	*CTA GCT CTA AAA CGT GGC GCA TTG TTC ACT GGA ACG T*
pNG572 Vps5_N-check_F	*GCCTTATTTCTGGGGTAATTAATCAGCG*
pNG609 Pep4 KO Guide2 F	*AGAAGTACCAGTATCGATGGGTTTTAGAG*
pNG610 Pep4 KO Guide2 R	*CTAGCTCTAAAACCCATCGATACTGGTACTTCTACGT*
pNG611 pep4 KO oligo F	*GTATTTAATCCAAATAAAATTCAAACAAAAACCAAAACTAACG*
	CTAAACTTTTCTTACTTCTCCGCCCTATCCTTTTCTGCC
pNG612 pep4 KO oligo R	*GGCAGAAAAGGATAGGGCGGAGAAGTAAGAAAAGTTTAGCG*
	TTAGTTTTGGTTTTTGTTTGAATTTTATTTGGATTAAATAC
pNG613 pep4KOctrl-F	*GAGAAGCCTACCACGTAAGGGAAG*
pNG614 pep4KO ctrl R	*GACATTATGGGCAGCAGCATAGAAC*

Uncropped gels and blot scans for supplementary figures

Supplementary Figure 1B

Raw SDS-PAGE gel showing the purity and integrity of ctRetromer and the subcomplex.

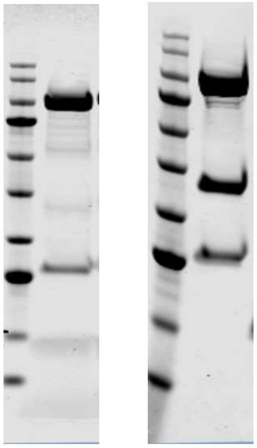

Supplementary Figure 5A.

Raw mass photometry diagram (in 40 mM NaCl buffer).

ctRetromer

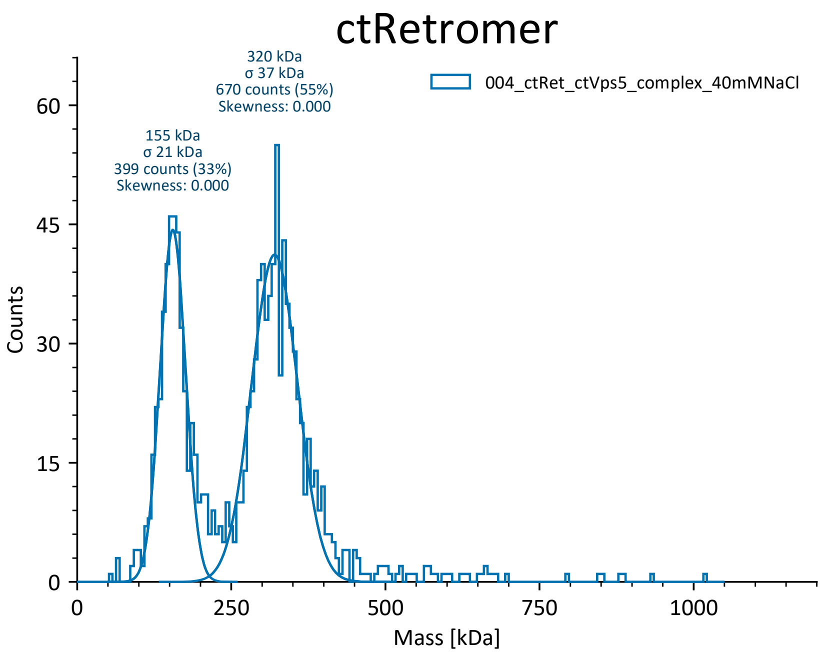

ctVps5

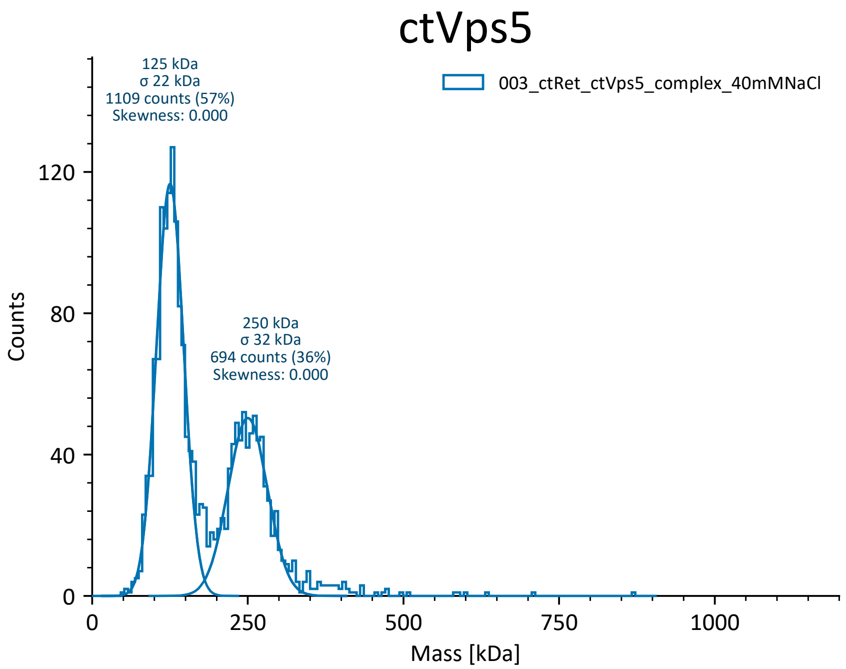

ctRetromer – ctVps5 complex

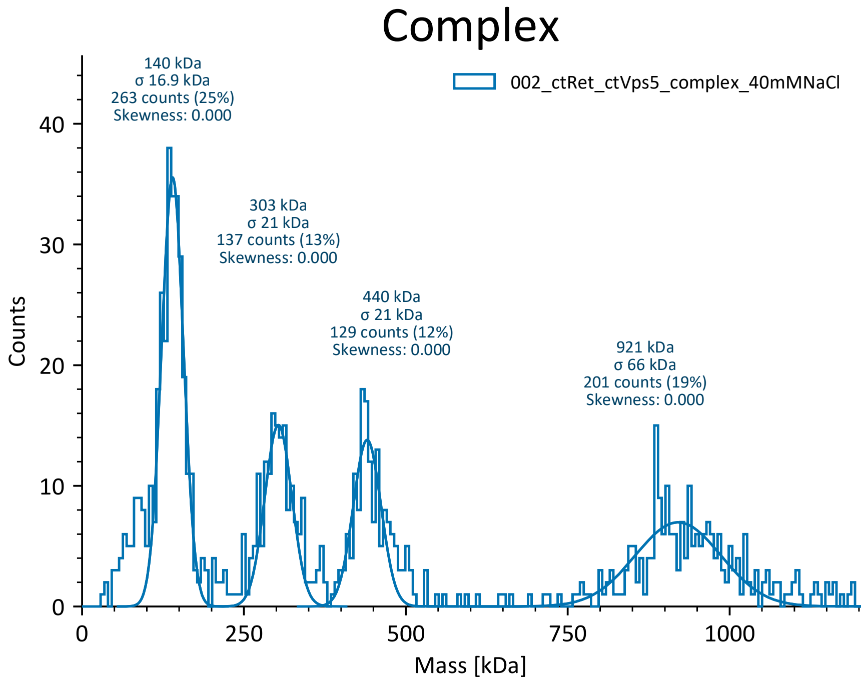

Supplementary Figure 6A.

Raw SDS-PAGE gel of liposome-pelleting assay of ctVps26 and ctVps5 in the presence of Folch I lipids supplemented with PtdIns(3)*P*.

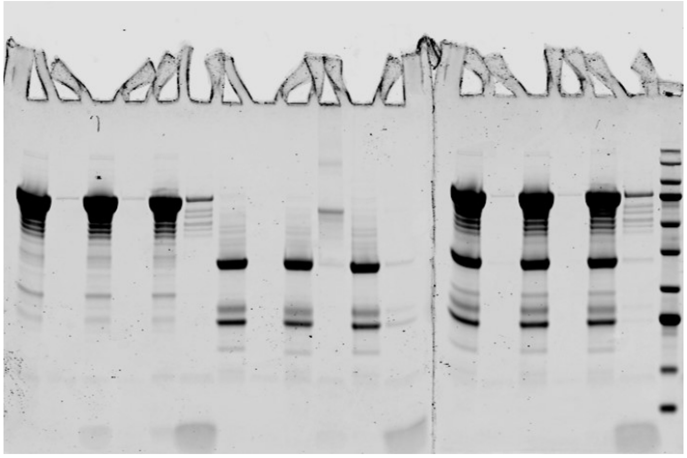

Supplementary Figure 10E.

Raw SDS-PAGE gel and uncropped blot of GST pull-down showing the weak interaction between GST-tagged scVps5$_{70\text{-}126}$ LF motif containing loop and hRetromer.

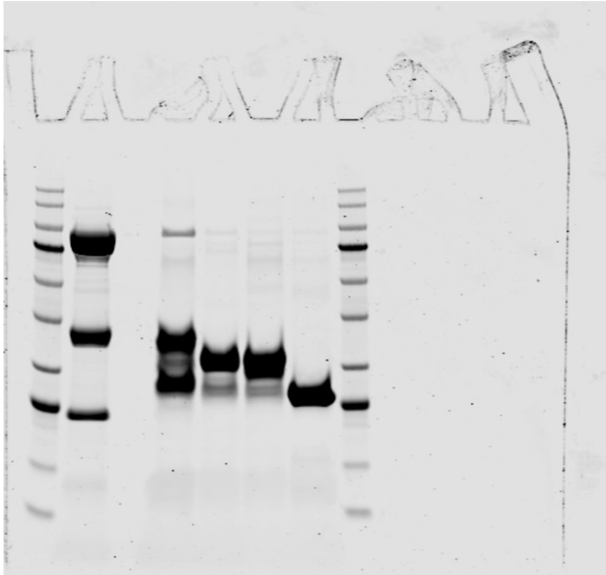

Raw blot

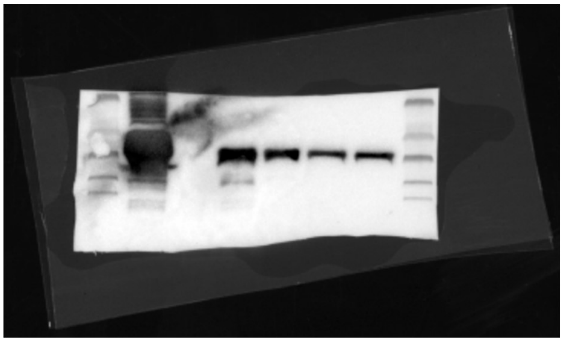

Supplementary Figure 14A

Raw mass photometry diagram of ctVps17 (in 200 mM NaCl buffer).

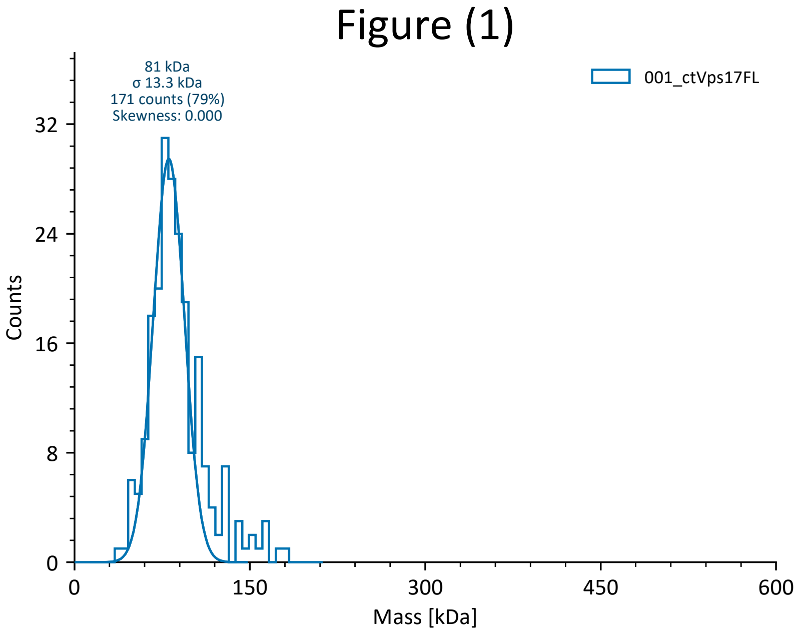

Raw SDS-PAGE gel of liposome pelleting assay of ctVps17.

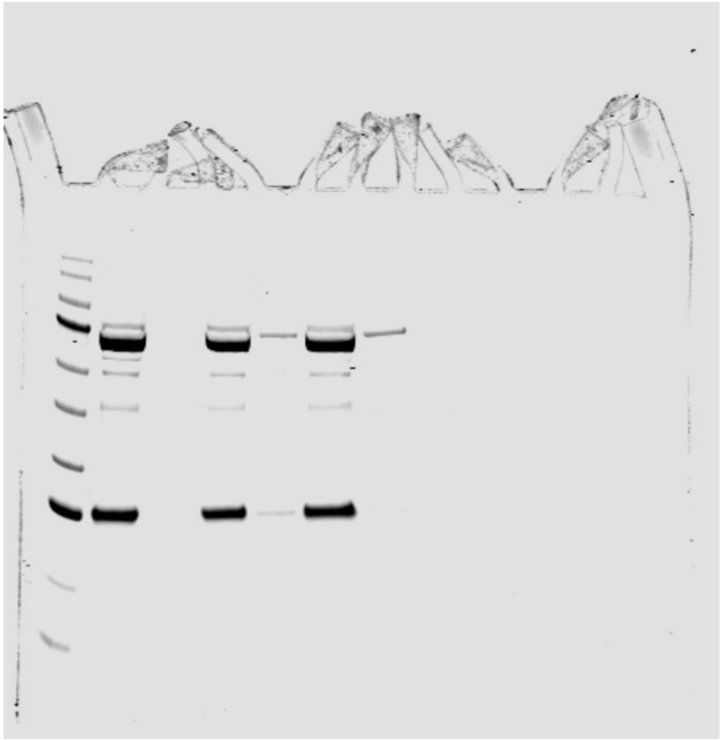

Supplement: Supplementary file 1 — Supplementary Information [file 41467_2025_58846_MOESM1_ESM.pdf]
