# Supplementary material for: Molecular basis for the assembly of the Vps5-Vps17 SNX-BAR proteins with Retromer
Source: Nat Commun. 2025 Apr 15;16:3568. doi: 10.1038/s41467-025-58846-8 (PMC12000511; doi:10.1038/s41467-025-58846-8)
Supplement: Supplementary file 2 — Description of Additional Supplementary Files [file 41467_2025_58846_MOESM2_ESM.pdf]

Description of Additional Supplementary Files

File name: Supplementary Movie 1
Description: (related to Fig. 5F). CryoET image of ctRetromer–Vps5–Vps17 coated membrane tubules.

File name Supplementary Movie 2
Description: (related to Fig. 5G). Reconstructed tomogram of ctRetromer–Vps5–Vps17 coated tubule shown along the tubule axis.

File name Supplementary Movie 3
Description: (related to Fig. 5G). Reconstructed tomogram of ctRetromer–Vps5–Vps17 coated tubule shown from the side.

File name: Supplementary Movie 4
Description: (related to Fig. 6A). Recruitment of scSNX-BAR-GFP and scRetromer-mRuby on supported membrane tubes in the presence of the inhibitory RT-D3 cyclic peptide.

File name: Supplementary Movie 5
Description: (related to Fig. 6A). Recruitment of scSNX-BAR-GFP and scRetromer-mRuby on supported membrane tubes in the presence of the control RT-D3 scrambled cyclic peptide.

File name: Supplementary Movie 6
Description: (related to Fig. 6F). Supported membrane tubes with 100 nM scSNX-BARs and and 300 μM of the cyclic peptide RT-D3 or RT-D3 scrambled.

File name: Supplementary Movie 7
Description: (related to Fig. S13A). Supported membrane tubes with 25 nM scRetroer-mClover and 25 nM scVps17 – scVps5 wild-type.

File name: Supplementary Movie 8
Description: (related to Fig. S13A). Supported membrane tubes with 25 nM scRetroer-mClover and 25 nM scVps17 – scVps5 quadruple mutant.
