# Supplementary material for: Molecular basis for the assembly of the Vps5-Vps17 SNX-BAR proteins with Retromer
Source: Nat Commun. 2025 Apr 15;16:3568. doi: 10.1038/s41467-025-58846-8 (PMC12000511; doi:10.1038/s41467-025-58846-8)
Supplement: Supplementary file 11 — Reporting Summary [file 41467_2025_58846_MOESM11_ESM.pdf]

nature portfolio

Corresponding author(s): Brett Collins

Last updated by author(s): 13 September 2024

Reporting Summary

Nature Portfolio wishes to improve the reproducibility of the work that we publish. This form provides structure for consistency and transparency in reporting. For further information on Nature Portfolio policies, see our Editorial Policies and the Editorial Policy Checklist.

Statistics

For all statistical analyses, confirm that the following items are present in the figure legend, table legend, main text, or Methods section.

n/a	Confirmed	
☒	☐	The exact sample size (n) for each experimental group/condition, given as a discrete number and unit of measurement
☒	☐	A statement on whether measurements were taken from distinct samples or whether the same sample was measured repeatedly
☒	☐	The statistical test(s) used AND whether they are one- or two-sided<br>*Only common tests should be described solely by name; describe more complex techniques in the Methods section.*
☒	☐	A description of all covariates tested
☒	☐	A description of any assumptions or corrections, such as tests of normality and adjustment for multiple comparisons
☒	☐	A full description of the statistical parameters including central tendency (e.g. means) or other basic estimates (e.g. regression coefficient) AND variation (e.g. standard deviation) or associated estimates of uncertainty (e.g. confidence intervals)
☒	☐	For null hypothesis testing, the test statistic (e.g. F, t, r) with confidence intervals, effect sizes, degrees of freedom and P value noted<br>*Give P values as exact values whenever suitable.*
☒	☐	For Bayesian analysis, information on the choice of priors and Markov chain Monte Carlo settings
☒	☐	For hierarchical and complex designs, identification of the appropriate level for tests and full reporting of outcomes
☒	☐	Estimates of effect sizes (e.g. Cohen's d, Pearson's r), indicating how they were calculated

Our web collection on statistics for biologists contains articles on many of the points above.

Software and code

Policy information about availability of computer code

Data collection

CryoET
Imaging acquisition was performed on a 200 kV Talos Arctica (Thermo Fisher Scientific) transmission electron microscope fitted with a Falcon 3EC direct electron detector (Thermo Fisher Scientific) at the University of New South Wales, Australia. A total of 73 dose-symmetric tilt series were collected with tilt range ± 60°, 3° angular increment and defoci between -3.5 µm and -5.0 µm. The acquisition magnification was 45,000x with a pixel size of 3.24 Å. Total dose of each tilt series was ranged from 120 - 130 e/A2. Assembly of tomograms from raw movies was done with IMOD 4.12.45 package82. Gain-reference corrected movies from the microscope were aligned, summed and filtered according to the deposited electron dose using aligframes (IMOD 4.12.45) to generate sorted tilt series used for tomogram reconstruction. Per-tilt defocus estimation was done on non-dose weighted stacks in ctfplotter 83. The stacks further aligned using fiducials-based, 2D-CTF corrected and used for weighted back projection tomogram reconstruction by etomo package (IMOD 4.12.45). For visualisation only, 8-times binned tomograms were processed with deep learning denoising and wedge restoration package IsoNet 84.

Xray crystallography
Data collected from the Australian Synchrotron were indexed and integrated by AutoXDS and scaled using Aimless75. Both structures were solved by molecular replacement using Phaser76 with native mVps29 structure as the initial model template. Both structures contain two copies of mVps29 in asymmetric unit with the electron density of peptide clearly visible. The refinement was performed using Phenix with inspection of resulting model in Coot guided by Fo–Fc difference maps77. Molprobity was used to evaluate the quality geometry of the refined structure78. Data collection and refinement statistics are summarized in Table S1. Molecular figures were generated using PyMOL and structural comparison was performed using DALI and Foldseek79,80. Sequence conservation was mapped on the structure with ConSurf server81.

Differential Scanning Fluorimetry

Thermal unfolding experiments were carried out to confirm the protein constructs applied in this study were properly folded or not. In brief, 0.2 mg/ml of purified ctVps26, ctVps29, ctVps29 – ctVps35, ctRetromer, ctVps5, and ctVps5 – ctVps17PX-BAR was prepared with the addition of 5X final concentration of SYPRO orange dye (Life Science). The sample was loaded into the 96-well plate and the relative fluorescence units were measured from 25°C to 85°C using the ROX dye calibration setting at 1°C increments using a ViiA7 real-time PCR instrument (Applied Biosystems). Experiments were performed with four replicates, and Tm was calculated using Boltzmann sigmoidal in Prism version 10.2.0 (GraphPad Software).

Data analysis: N/A

For manuscripts utilizing custom algorithms or software that are central to the research but not yet described in published literature, software must be made available to editors and reviewers. We strongly encourage code deposition in a community repository (e.g. GitHub). See the Nature Portfolio guidelines for submitting code & software for further information.

Data

Policy information about availability of data

All manuscripts must include a data availability statement. This statement should provide the following information, where applicable:

- Accession codes, unique identifiers, or web links for publicly available datasets
- A description of any restrictions on data availability
- For clinical datasets or third party data, please ensure that the statement adheres to our policy

Crystal structure of hVps29 – ctVps571-80 peptide complex has been deposited at the Protein Data Bank with accession codes (8FUD). All the relevant raw data related to this study is available from the corresponding authors on request.

Research involving human participants, their data, or biological material

Policy information about studies with human participants or human data. See also policy information about sex, gender (identity/presentation), and sexual orientation and race, ethnicity and racism.

Reporting on sex and gender: N/A

Reporting on race, ethnicity, or other socially relevant groupings: N/A

Population characteristics: N/A

Recruitment: N/A

Ethics oversight: N/A

Note that full information on the approval of the study protocol must also be provided in the manuscript.

Field-specific reporting

Please select the one below that is the best fit for your research. If you are not sure, read the appropriate sections before making your selection.

☒ Life sciences ☐ Behavioural & social sciences ☐ Ecological, evolutionary & environmental sciences

For a reference copy of the document with all sections, see nature.com/documents/nr-reporting-summary-flat.pdf

Life sciences study design

All studies must disclose on these points even when the disclosure is negative.

Sample size: N/A

Data exclusions: N/A

Replication: N/A

Randomization: N/A

Blinding: N/A

Reporting for specific materials, systems and methods

We require information from authors about some types of materials, experimental systems and methods used in many studies. Here, indicate whether each material, system or method listed is relevant to your study. If you are not sure if a list item applies to your research, read the appropriate section before selecting a response.

Materials & experimental systems

n/a	Involved in the study
☒	☐ Antibodies
☐	☒ Eukaryotic cell lines
☒	☐ Palaeontology and archaeology
☒	☐ Animals and other organisms
☒	☐ Clinical data
☒	☐ Dual use research of concern
☒	☐ Plants

Methods

n/a	Involved in the study
☒	☐ ChIP-seq
☒	☐ Flow cytometry
☒	☐ MRI-based neuroimaging

Eukaryotic cell lines

Policy information about cell lines and Sex and Gender in Research

Cell line source(s)	Strain Genotype<br>SEY6210 Δvps5 Vam10-V5::LEU2 Vps10mNeonGreen<br>SEY6210 Δvps5 Vam10-V5::LEU2 Vps10mNeonGreen , plasmid pRS314-VPS5-HA::TRP1<br>SEY6210 Δvps5 Vam10-V5::LEU2 Vps10mNeonGreen , plasmid pRS314-VPS5Δ1-280-HA::TRP1<br>SEY6210 Δvps5 Vam10-V5::LEU2 Vps10mNeonGreen , plasmid pRS314-VPS5L196R-HA::TRP1<br>SEY6210 Δvps5 Vam10-V5::LEU2 Vps10mNeonGreen, plasmid pRS314-VPS5L196R L121G F122G F161E-HA::TRP1
Authentication	N/A
Mycoplasma contamination	N/A
Commonly misidentified lines<br>(See ICLAC register)	*Name any commonly misidentified cell lines used in the study and provide a rationale for their use.*

Plants

Seed stocks	N/A
Novel plant genotypes	N/A
Authentication	N/A
