# Supplementary material for: Molecular basis for the assembly of the Vps5-Vps17 SNX-BAR proteins with Retromer
Source: Nat Commun. 2025 Apr 15;16:3568. doi: 10.1038/s41467-025-58846-8 (PMC12000511; doi:10.1038/s41467-025-58846-8)

**Figure 2C**

Raw mass photometry diagram (in 200 mM NaCl buffer).

ctvps5

ctRetromer

ctRetromer – ctVps5 complex

ctRetromer – ctVps5 complex + RT-D3 cyclic peptide

**Figure 2D.**

ctVps5 control in the presence of DMSO, RT-D3 or RT-L4.


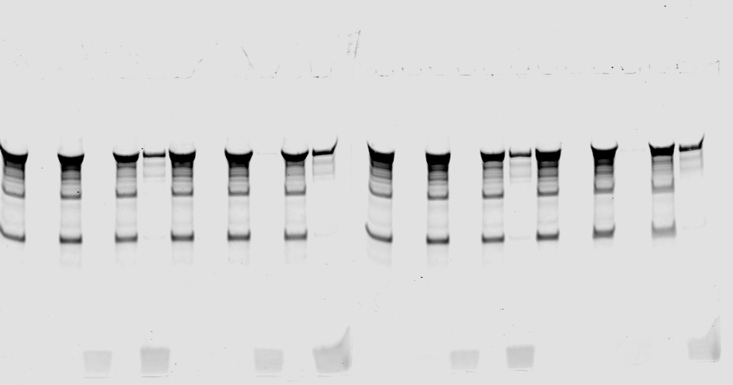


ctRetromer – ctVps5 complex in the presence of DMSO, RT-D3 or RT-L4.


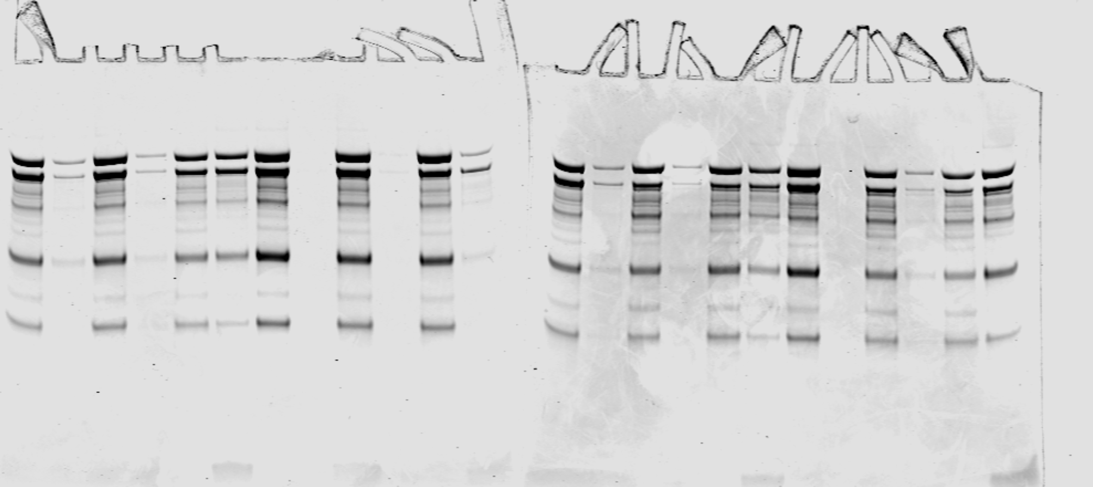


**Figure 5B.**

Raw SDS-PAGE gel of the GST pull-down demonstrating the direct interaction between GST-ctVps5-ctVps17_PX-BAR_ and ctRetromer.


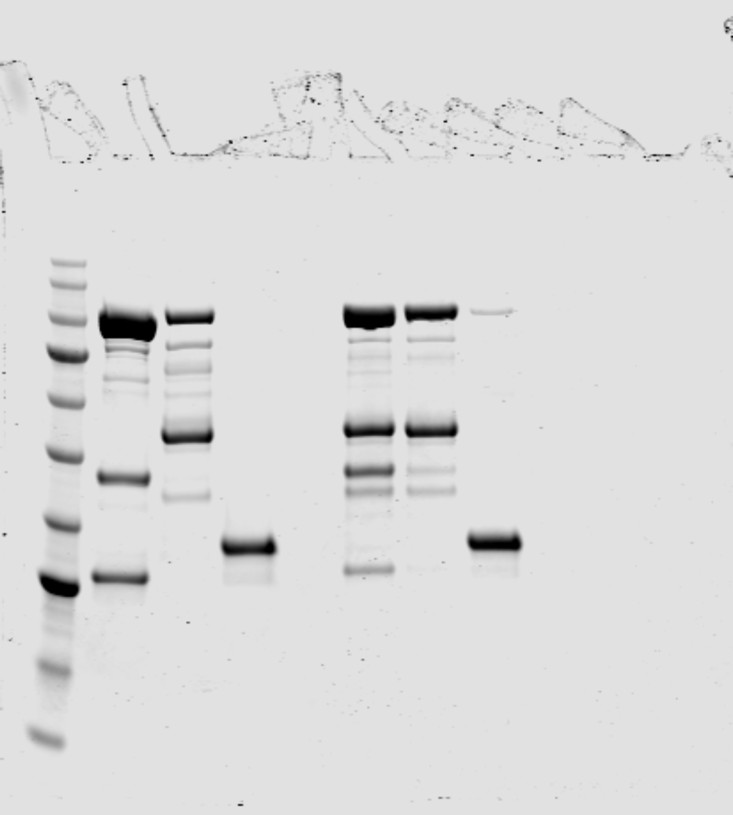


**Figure 5C.**

Raw mass photometry diagram and the SDS-PAGE gel of heteropentameric complex.


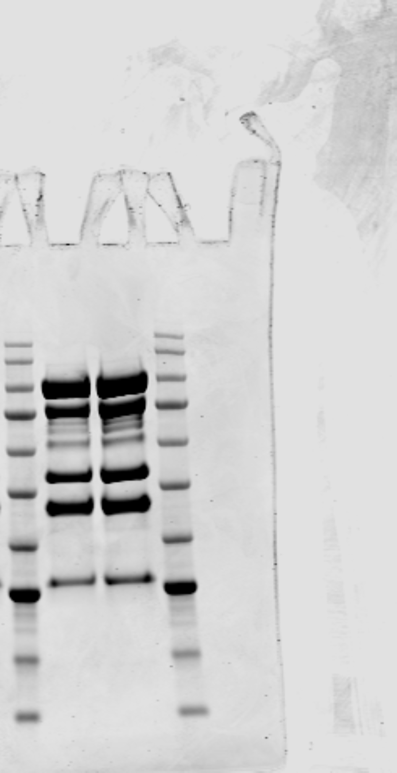


**Figure S1B.**

Raw SDS-PAGE gel showing the purity and integrity of ctRetromer and the subcomplex.


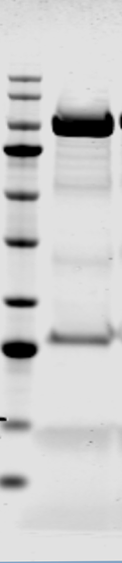

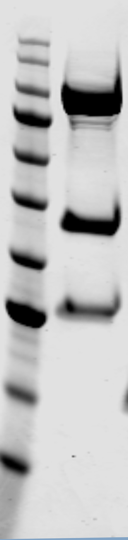


**Figure S5A.**

Raw mass photometry diagram (in 40 mM NaCl buffer).

ctRetromer

ctVps5

ctRetromer – ctVps5 complex

**Figure S6A.**

Raw SDS-PAGE gel of liposome-pelleting assay of ctVps26 and ctVps5 in the presence of Folch I lipids supplemented with PtdIns(3)*P*.


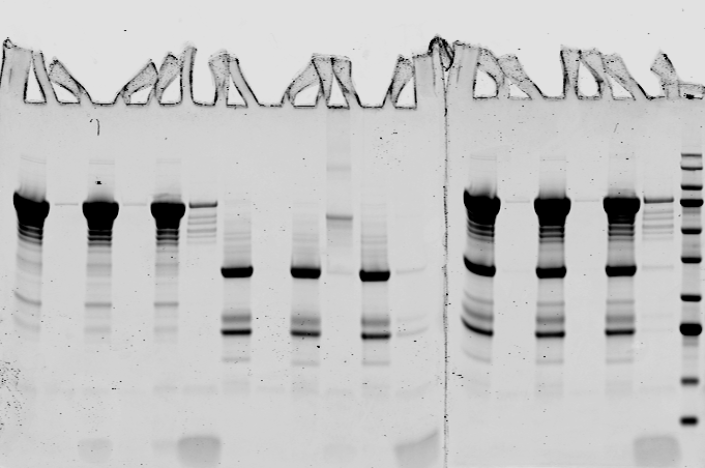


**Figure S10E.**

Raw SDS-PAGE gel and uncropped blot of GST pull-down showing the weak interaction between GST-tagged scVps5_70-126_ LF motif containing loop and hRetromer.


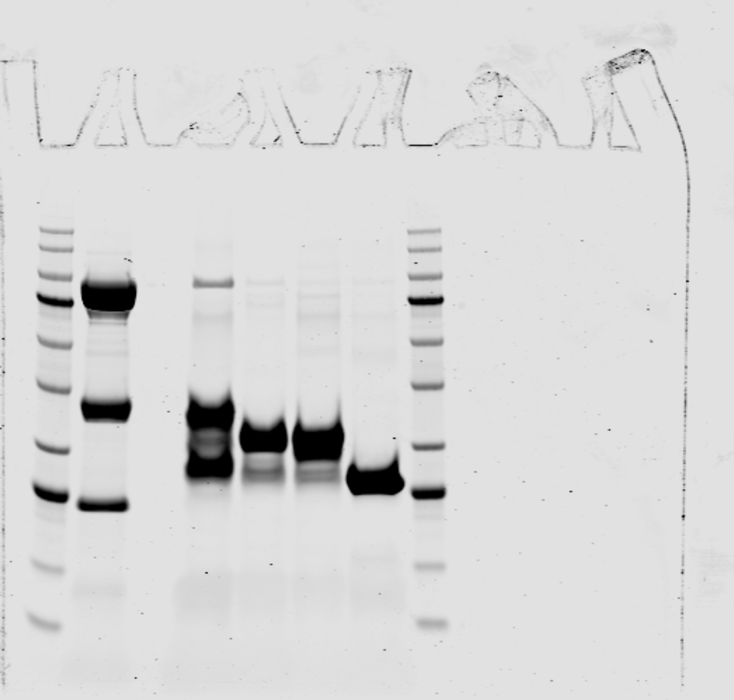


Raw blot


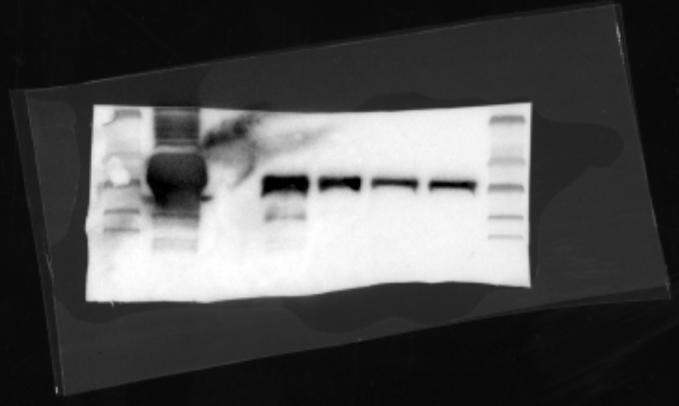


**Figure S14A**

Raw mass photometry diagram of ctVps17 (in 200 mM NaCl buffer).

**Figure S14B**

Raw SDS-PAGE gel of liposome pelleting assay of ctVps17.


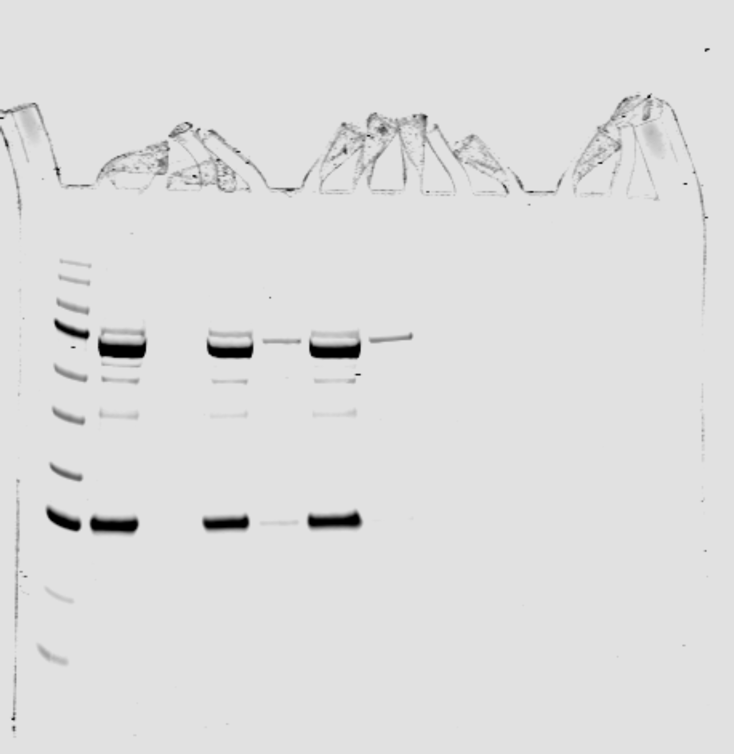

Supplement: Supplementary file 13 — Source data [file 41467_2025_58846_MOESM13_ESM.zip › Uncropped data for Gels and Blots and mass photometry.docx]
